# PDE3 is the major cGMP-PDE in adult mouse ventricular cardiomyocytes

**DOI:** 10.1186/2050-6511-14-S1-P26

**Published:** 2013-08-29

**Authors:** Konrad R Götz, Viacheslav O Nikolaev

**Affiliations:** 1 Department of Cardiology and Pneumology, Georg August University Medical Center, University of Göttingen, D-37083 Göttingen, Germany

## Background

cGMP is an important second messenger which is involved in the regulation of cardiac contractility and pathological hypertrophy. Signaling by cGMP is considered cardioprotective, but little is known about the spatio-temporal dynamics of cGMP and its regulation in adult cardiomyocytes.

## Materials and methods

We generated a transgenic mouse model with cardiomyocyte-specific expression of the highly sensitive fluorescence resonance energy transfer (FRET)-based cGMP biosensor red cGES-DE5. This sensor mouse allows us to performed FRET measurements of cGMP in freshly isolated adult mouse ventricular myocytes, to visulaize real-time dynamics of cGMP. To analyze cAMP and cGMP interactions (cGMP/cAMP signaling crosstalk), FRET experiments were performed in cardiomyocytes isolated from mice, transgenically expressing the FRET-based cAMP sensor Epac1-camps.

## Results

In adult mouse cardiac myocytes, cytosolic cGMP levels are low (~10 nM), but can be markedly increased by stimulation of particulate guanylyl cyclases (GC-A, GC-B) with natriuretic peptides (CNP>>ANP/BNP). In contrast, stimulation of the soluble guanylyl cyclase (sGC) with NO-donors such as SNAP had no effect. However, constitutive activity of this cyclase is involved in basal cGMP production, since stimulating cardiomyocytes with the sGC inhibitor ODQ showed a decrease of basal cGMP levels. This basal cGMP production is balanced by phosphodiesterase (PDE) activity. Inhibiting PDE1,2 and 5 had only little effect, whereas PDE3 represents the major cGMP-PDE family controlling cGMP levels at basal state, in the presence of cGMP agonists (such as SNAP, ANP, and CNP) and in a model of cardiac hypertrophy (transverse aortic constriction model). In addition we could show that cGMP pools produced by GC-B after CNP stimulation are mainly regulated by PDE3, so that the receptor and this PDE form one functional unit important for the regulation of cGMP/cAMP cross-talk and modulation of cAMP levels.

## Conclusion

We conclude that PDE3 is the dominant PDE regulating cGMP.

